# *In vitro* induction of *Entamoeba gingivalis* cyst-like structures from trophozoites in response to antibiotic treatment

**DOI:** 10.3389/fcimb.2023.1201394

**Published:** 2023-07-04

**Authors:** Christin Becker, Aysegül Adam, Henrik Dommisch, Thomas Stach, Arne S. Schaefer

**Affiliations:** ^1^ Molecular Genetics of Oral Inflammatory Diseases Group, Institute for Dental and Craniofacial Sciences, Department of Periodontology, Oral Medicine and Oral Surgery, Charité – University Medicine Berlin, Corporate Member of Freie Universität Berlin, Humboldt-Universität zu Berlin, and Berlin Institute of Health, Berlin, Germany; ^2^ Department of Molecular Parasitology, Institute of Biology, Humboldt-Universität zu Berlin, Berlin, Germany

**Keywords:** *Entamoeba gingivalis*, cysts, encystation, periodontitis, periimplantitis, antibiotics

## Abstract

**Background:**

Entamoeba *gingivalis (E. gingivalis)* is an anaerobic protozoan that is strongly associated with inflamed periodontal pockets. It is able to invade the mucosal epithelium of the human host, where it can feed on epithelial cells and elicit a severe innate immune response. Unlike other *Entamoeba* species, it is considered that *E. gingivalis* cannot form cysts, because it is a non-infectious protozoan. The lack of encystation capability would make it susceptible to periodontal treatment. However, it is not clear how the human host becomes infected with *E. gingivalis* trophozoites. We investigated the ability of *E. gingivalis* to encapsulate in response to an unfavorable environment *in vitro*.

**Methods:**

Different strains of *E. gingivalis*, isolated from inflamed periodontal pocket samples, were cultured for 8 days in the presence or absence of the antimicrobials amoxycillin and metronidazole. To reveal cyst formation, we investigated the morphology and ultrastructure of the amoeba by light, fluorescence, transmission and scanning electron microscopy. We also used the fluorescent dye calcofluor white M2R to demonstrate chitin present in the cyst wall.

**Results:**

We observed exocysts and an intra-cystic space separating the encapsulated trophozoite from the environment. Remarkably, cysts showed a smooth surface, polygonal edges and smaller size compared to free-living trophozoites. In addition, encapsulated trophozoites that detached from the cyst wall had a dense cytoplasma without phagocytic vesicles. The cyst walls consisted of chitin as in other *Entamoba* species. The encapsulated trophozoids were mononuclear after antibioticinduced encapsulation.

**Discussion:**

We conclude that *E. gingivalis* cyst formation has significant implications for dissemination and infection and may explain why established treatment approaches often fail to halt periodontal tissue destruction during periodontitis and peri-implantitis.

## Introduction

*Entamoeba gingivalis* (*E. gingivalis*) is a parasitic protozoan whose active form (known as trophozoite) colonizes the human oral cavity and is found in up to 80% of inflamed periodontal pockets from patients with severe periodontitis (Bonner et al., 2014; Bao et al., 2020), a complex inflammatory disease of the oral cavity (Eke et al., 2012; Marcenes et al., 2013; Eke et al., 2015). Characteristic for periodontitis is long-term and recurrent inflammation of periodontal pockets, which is considered to result from a polymicrobial insult from as yet unidentified microorganisms (Scannapieco and Dongari-Bagtzoglou, 2021). Necrotizing ulceration of the interdental papilla and the periodontal and alveolar ligament also characterize some forms of periodontitis (Caton et al., 2018). Mucosal bleeding and tissue destruction is also characteristic for clinical forms of amoebiasis, where *Entamoeba histolytica* (*E. histolytivca*) infects the colonic epithelium. Comparable to the effects of *E. histolytica*, infection of gingival epithelial cells by *E. gingivalis* also causes a significant induction of proinflammatory cytokine signaling with high levels of expression of interleukin-8, the main cytokine that activates and attracts neutrophils to the inflammatory region (Kim et al., 1998; Bao et al., 2020; Bao et al., 2021). The pathogenic potential of *E. gingivalis* is also implied by the observations that similar to *E. histolytica*, it is able to invade the mucosal epithelium, where it kills epithelial cells by trogocytosis, a process in which it physically extracts and ingests cellular material from human epithelial cells. Scanning electron micrograph (SEM) of *E. histolytica* and *E. gingivalis* amoebae show that trogocytosis of oral epithelial cells by *E. gingivalis* looks highly similar to trogocytosis of colon epithelial cells by *E. histolytica* (Ralston et al., 2014; Bao et al., 2021). Likewise, it was shown that trogocytosis by *E. histolytica* and *E. gingivalis* contributes to cell killing and tissue invasion (Ralston et al., 2014) (Bao et al., 2021). However, despite of this evidence, *E. gingivalis* is currently being considered commensal and not infectious (Bonner et al., 2018). A significant argument for this consideration is that essential for infection and the epidemiological impact of *E. histolytica* is its capability to form cyst walls. These structures are composed of chitin, which are hard and impermeable to small molecules and protect the parasite from natural environmental insults outside of the colon, such as environmental oxygen, drying, osmotic shock in water, or lysis by stomach acids and duodenal proteases. Due to their wall organization with a high content of chitin, amoeba cysts also confer resistance to various antibiotic medication and therefore represent a serious problem in the treatment of amoebic infections. In contrast to *E. histolytica* and other anaerobic *Entamoeba* species, cyst formation of *E. gingivalis* has not yet been observed. However, it is unclear how the human host is infected by facultatively anaerobic E. gingivalis trophozoites. Oral infection with anaerobic trophozoites *via* water or food is hard to imagine One possibility would be infection through contaminated saliva, but this would be a very limited and inactive way to reproduce or respond to an unfavorable environment. No loss of encystation ability was observed in other human protozoan parasites either. Therefore, we hypothesized that *E. gingivalis*, similar to *E. histolytica*, is capable of encystation when environmental conditions become unfavorable. In addition, cyst formation would imply the infectious potential of this parasite and the ability to resist some therapeutically relevant approaches. Here, we report the *in vitro* encystation of *E. gingivalis* trophozoites induced by the presence of antibiotics in the culture medium. Compared to trophozoites, mononuclear cysts were smaller, spherical, had smooth surfaces, polygonal edges, and the presence of chitin as part of the cyst wall.

## Materials and methods

### Culture of *E. gingivalis* and induction of cyst-like structures

Subgingival plaque from inflamed periodontal pockets of periodontitis patients was collected with curettes as part of periodontal therapy at the Department of Periodontology, Oral Medicine and Oral Surgery, Charité - University Medicine, Berlin. Immediately after subgingival plaque collection, samples were transferred in 30 mm x 15 mm petri dishes containing 2 ml TYGM-9 medium (ATCC) and covered with 1ml Nujol mineral oil and cultured under anaerobic conditions at 35°C. After 48 hours, the presence of *E. gingivalis* in the anaerobic plaque cultures was examined visually by light microscopy (Leitz Labovert, 16x, 0.4) and by PCR. Purification of *E. gingivalis*’s DNA was isolated directly from growth medium using phenol chloroform extraction (Rosenbaum et al., 2019). PCR was performed using primers designed for the identification of *E. gingivalis* (*E. gingivalis*-fwd: AGG AAT GAA CGG AAC GTA CA, *E. gingivalis*-rev: CCA TTT CCT TCT TCT ATT GTT TCA C (203 bp product size) (Bonner et al., 2014), as previously performed (Bao et al., 2020). Primers for amplification of DNA sequences of the human Actin gene were used as positive control for DNA isolation Actin-fwd: ATT TAG CGC CAA TTC CCA, Actin-rev: GGC GGG GTC TTT GTC TGA, 122 bp product size). The PCR program was as follows: initial denaturation (94°C, 3′30″), 40 cycles (94°C, 1′; 60°C, 1′; 72°C, 1′), final extension (72°C, 1′). We detected the presence of *E. gingivalis* in ~3 out of 4 plaque samples that we received from periodontal inflamed gingival pockets with > 4 mm probing depth. However, after 2 days of *in vitro* cultivation, only 10% of plaque samples showed moving trophozoites observed under the light microscope. We used only those cultures with both, observed moving trophozoites and a positive PCR test for further experiments. Cultures that contained *E. gingivalis* trophozoites were subsequently incubated with or without amoxycillin (13.7 µg/ml) and metronidazole (2.5 µg/ml) for 8 days as described in (Goodson, 2000). These antibiotics and concentrations corresponded to the therapeutic application of the “van Winkelhoff-Cocktail”, an antibiotic combination that is often prescribed in periodontal therapy (van Winkelhoff et al., 1989). Seven independent experiments were carried out for antibiotic treatment and antibiotic free-cultures and induction of cyst formation. The local ethical committee approved to the use of plaque samples from periodontitis patients to culture *E. gingivalis* (EA1/169/20) and informed patient consent was obtained.

### Microscopy and size estimation of trophozoites and cysts

*E. gingivalis* cells were stained with Lugol’s iodine in a 1:1 dilution with 25% glacial acetic acid (Carl Roth). The morphological structures of the specimen were visualized using light microscopy (Leica DM750 with FlexaCam C3). To obtain a size range for the diameter of trophozoites, we used a scale bar and measured the trophozoites lengths (N = 26) at the same culture age (2 days). For fluorescence microscopy, *E. gingivalis* cells were fixed with 2.5% Glutaraldehyde for 30 min and washed 3 times in 1xPBS. After centrifugation (300g, 5 min), *E. gingivalis* cells were placed onto microscope slides and stained with calcofluor white M2R (Sigma-Aldrich), a fluorescent dye that specifically binds to components of chitin. Fluorescence microscopy was performed with laser scanning confocal microscope LSM 700 (Zeiss).

For transmission electron microscopy (TEM), *E. gingivalis* cells were fixed and dehydrated as described in (Gluenz et al., 2015). In brief, trophozoites were fixated in 2.5% glutaraldehyde in 0.1 M Cacodylat buffer (1% NaCl, pH 7,2) for 30 minutes. Washing was performed 3x in 1 x PBS at 300g for 10 minutes.

Subsequently, 2% agarose (in 1x PBS) was melted at 60°C (freshly prepared: 95°C) and kept at 37°C. The solution containing the primary-fixed cells was centrifuge, the supernatant discarded and the pellet kept at 37°C. The warm agarose was pipetted onto the pellet (8 agarose beads per 60µl) and carefully resuspended with the pipette at 37°C. The suspension was transferred dropwise onto Parafilm and solidified in the refrigerator in a humidity chamber. The resulting agarose beads containing the amoeba were immediately processed further to avoid drying. Secondary fixation was performed in 1% Osmiumtetroxid (OsO_4_) in 1xPBS for 30 minutes. Subsequently, coverslips were washed 2x with ddH_2_O and incubated in 0.5% UAc (in ddH_2_O) for 20 minutes and 1x washed in ddH_2_O. The samples were dehydrated with increasing ethanol concentrations (30%, 50%, 70%, 80%, 90% und 96% EtOH, 10 minutes each step, 2x 100% EtOH for 30 minutes). The specimens were first immersed in propylene oxide (PO, Electron Microscopy Sciences), 2x for 10 minutes and subsequently embedded in araldite (Agar Scientific Ltd.), stepwise increasing PO: araldite A (3:1, 2:1, 1:1 and 1:2, 1:3 for 2 h and 2.5 h, respectively, until 100% immersion (o/n) and araldite A was replaced with araldite B (4h) and subsequently hardened at 60°C for 24h. Ultrathin sections (60 nm) were cut (Ultracut UCT, Leica), placed on single slot grids and contrasted with uranyl acetate and lead citrate in an automatic stainer. Ultrathin sections were examined at 50 or 80 kV in a EM 900 (Zeiss) transmission electron microscope at 5000x to 12000x using a CCD camera (2K-Wide-angle) and the software ImageSNP (Tröndle).

For scanning electron microscopy (SEM), *E. gingivalis* cells were fixed in 2.5% glutaraldehyde (in 1xPBS, pH7.2) for 60 minutes and washed 5 x in 1xPBS for 5 min. After each step, the cell suspension was centrifuged in the falcon tube at 1800 rpm for 8 min, the supernatant was removed, and the cell pellet resuspended in fresh washing buffer. The trophozoites were transferred on poly-L-lysine coated coverslips (12mm, Corning BioCoat) as described in (Gluenz et al., 2015). The coverslips were distributed into 24 well plates. After the final wash of the 1st fixation, the cell suspension was centrifuged and the supernatant removed. The cell pellet was then resuspended in the remaining buffer and then dropped onto the coverslips in the well. The cells could then settle and adhere on to the coverslip (60 min). For the secondary fixation, the reagents were pipetted directly into the wells: OsO_4_ in 1x PBS pH 7.2 (30 min), followed by 3x ddH_2_O washing (5 min). Dehydration was performed as described for TEM. Subsequently, ethanol was exchanged with a 1:1 dilution of 100% EtOH and hexamethyldisilazan (HMDS, Carl Roth) for 30 min followed by incubation with pure HMDS for 30 minutes. Subsequently, HMDS was removed and the specimen were air-dried on filter paper, mounted on the coverslip with carbon planchet onto a sample stub and sputter coated (30 mA, 35 mm, 90 seconds) with 15 nm of gold in a SCD 005 sputter coater (BAL-TEC AG, Balzers, Liechtenstein). *E. gingivalis* cells were examined at 10 to 20kV in a LEO 1430 scanning electron microscope (Zeiss) using the software SmartSEM v.6 (Zeiss).

## Results

### Combined amoxycillin and metronidazole treatment induced formation of cyst-like structures from *E. gingivalis* trophozoites

**Figure 1:**
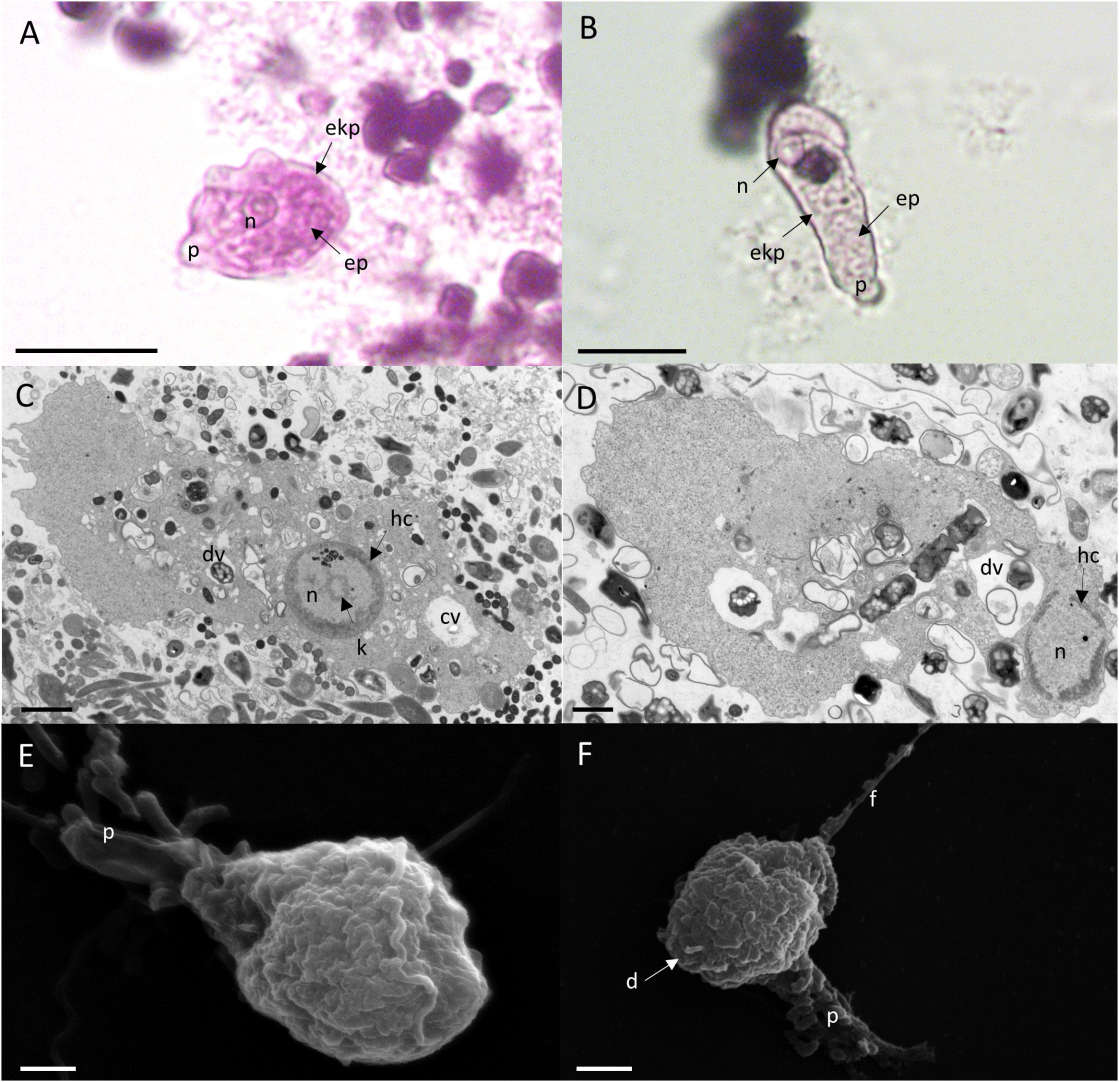
*E. gingivalis* trophozoites are characterized by granulose wrinkled walls, pseudopodia, numerous digestive vacuoles. Upper panel: Trophozoites under the light microscope show a distinct nucleus (n), pseudopodia (p), a granulose endoplasma (ep) and a transparent ektoplasma (ekp), which are located adjacent to the distinct plasma membrane as characteristic features **(A, B)**. Scale bar = 10 µm. Middle panel: TEM shows the characteristic features of the nucleus (n) and cytoplasma of a trophozoite. The nucleus has a circular karyosome (k), condensed peripheral heterochromatin (hc) and additionally shows a cluster of small round shaped electron dense nuclear bodies. In the cytoplasm, numerous digestive vacuoles (dv) and a contractile vacuole (cv) are visible **(C, D)**. Scale bar = 1 µm. Lower panel: SEM shows surface ultrastructure of trophozoites characterized by a granulose wrinkled membrane and smooth pseudopods (p), filipodes (f) and digipodes (d) **(E, F)**. Scale bar = 2 µm.

We cultured *E. gingivalis* from subgingival plaque collected from inflamed periodontal pockets from periodontitis patients under anoxic conditions. After 2 days of cultivation, the presence of *E. gingivalis* was observed by light microscopy and verified by PCR with *E. gingivalis*-specific primers. *E. gingivalis* containing cultures showed characteristic forms of mobile trophozoites (**Figures 1A, B**). The trophozoites generally exhibited an elongated shape and continuously formed one or more pseudopods at different positions during locomotion. The cytoplasma contained several food vacuoles and a contractile vacuole. Peripheral chromatin characterized the single nucleus. TEM additionally showed the characteristic circular karyosome with a central body and clusters of small, round electron-dense nuclear bodies (**Figures 1C, D**). SEM showed the presence of various pseudopods and filipodes, thin filaments of short and elongated forms (**Figures 1E, F**). The diameter of the trophozoites had sizes range of 10 – 25 µm (mean = 16 µm ± 5 µm, N = 26 trophozoites in a 2 days old culture). The trophozoites’ size range was identical to that of *E. histolytica* trophozoites, which have a size range of 10 – 27 µm when isolated from stool (Gleason et al., 1963).

**Figure 2:**
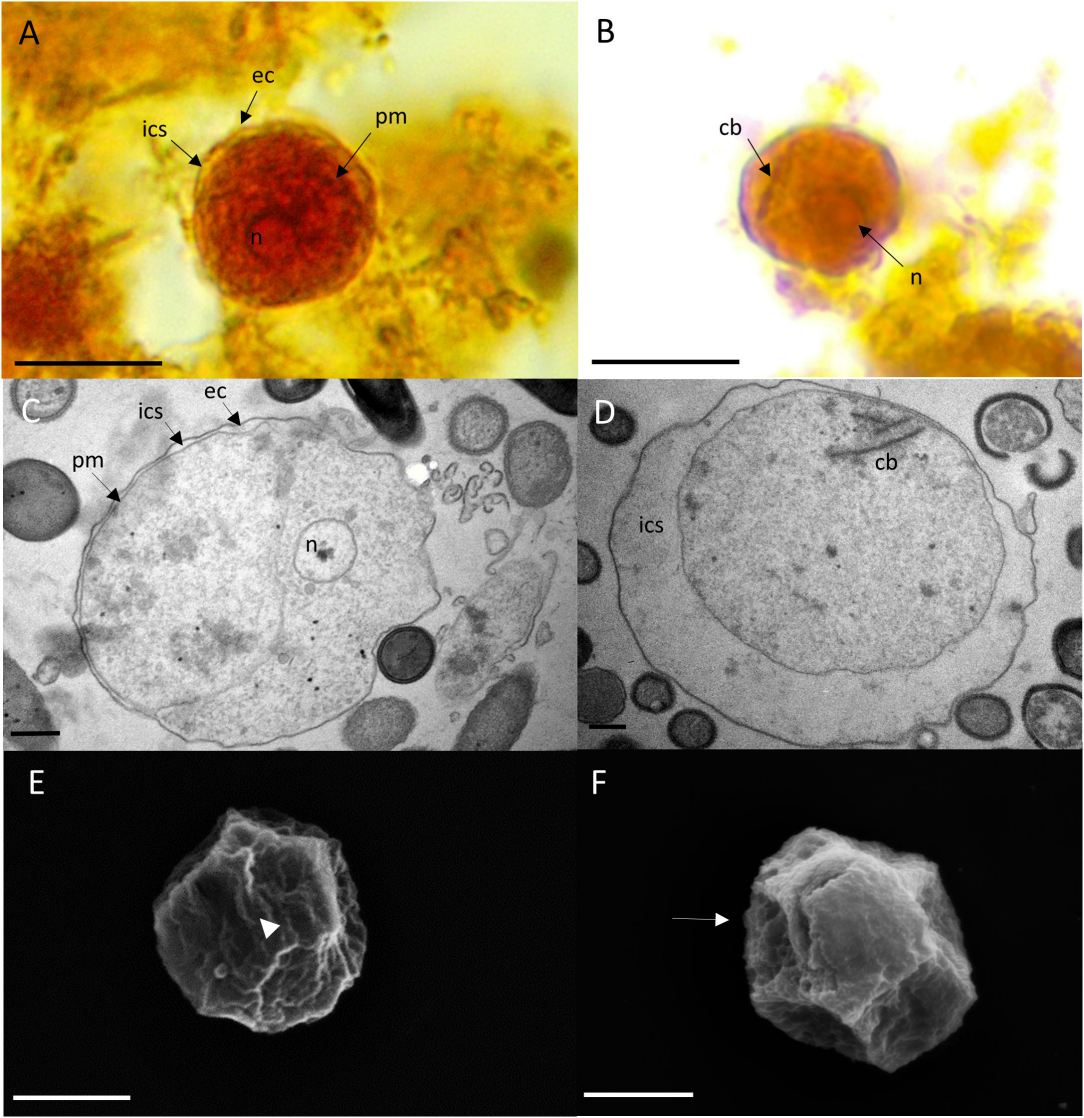
*E gingivalis* cysts are characterized by trophozoites that are detached from an encasing exocyst, condensed cytoplasma and smooth polygonal outer surfaces. Upper panel: Encysted trophozoites under the light microscope show a wrinkled plasma membrane (pm) separated from the encasing exocyst (ec) by a clear intercystic space (ics). The trophozoite has a single nucleus (n) **(A)**. A chromatoid body (cb) is visible outside of the nucleus **(B)** in some cells. Scale bar = 10 µm. Middle panel: TEM reveals characteristic features of mature cysts. The trophozoite’s plasma membrane is separated from the exocyst by an intra cystic space. The cytoplasma is homogeneous and does not contain digestive vacuoles. A compartment is more electron transparent and may contain glycogen **(C)**. The encysted trophozoite has a single nucleus and splintered chromatin bodies **(D)**. Scale bar = 1 µm. Lower panel: SEM reveals ultrastructure of polygonal cysts with a distinct ostiole (arrowhead) **(E)** and operculum (arrow) **(F)**. Scale bar = 2 µm.

After 2 days, the culture medium of the cultures containing *E. gingivalis* trophozoites was replaced with fresh medium supplemented with the antibiotics amoxycillin and metronidazole and incubation of the cultures continued. During the first 2 days of cultivation and after addition of amoxycillin and metronidazole, we could not observe any dividing trophozoites. After 8 days of cultures treated with amoxycillin and metronidazole, we observed cells with ultrastructural features of amoebic cysts (**Figure 2**). Under the light microscope (**Figures 2A, B**) and in TEM (**Figures 2C, D**) the size of the cysts was 7µm–13µm (mean = 11µm (± 2µm, N = 12), which was smaller than trophozoites. Smaller cyst sizes compared to trophozoites have also been reported for cysts from *E. invadens*, a reptilian amoebozoan parasite closely related to *E. histolytica* (Ehrenkaufer et al., 2013), with the cyst being about half the average diameter of the trophozoite (Singh et al., 2021). However, some cysts where even smaller, measuring ~4 µm, as documented by SEM (**Figures 2E, F**). These sizes are similar to those observed in *Hartmanella vermiformis* cysts. Remarkably, *E. gingivalis* cysts were smaller than *E. histolytica* cysts, the latter ranging in size from 10 - 20 µm (Aguilar-Diaz et al., 2010). Similar to *E. histolytica* cysts (Aguilar-Diaz et al., 2010), *E. gingivalis* cysts were smooth-surfaced and spherical in shape. While the cytoplasm of trophozoites contained numerous vesicles (**Figures 1C, D**), *E. gingivalis* cysts were faintly vesicular and appeared more compacted 8 days after antibiotic treatment (**Figures 2A-D**). This observation is similar to *E. invadens* cysts, which also show dense cytoplasm during the first 12 hours after encystation (Singh et al., 2021). However, older *E. invadens* cysts are vesicular and this is similar to *E. histolytica* cysts, in which the cytoplasm of mature cysts contains abundant vesicles (Chavez-Munguia et al., 2003). We therefore consider it possible that older *E. gingivalis* cysts are also more vesiculated.

Some cysts also showed a large, more electron- permeable compartment that may contain glycogen or lipids (**Figure 2C**), as described for *Entamoeba invadens* (Mousa et al., 2020) and *Acanthamoeba lugdunensis* (*A. lugdunensis*) cysts (Garajova et al., 2019), respectively. In some cells, we noted the presence of putative splintered chromatoid bodies (**Figure 2D**). For several *Entamoeba* species, chromatoid bodies have been described to arise in early cystic stages by aggregation of ribosomes forming dense crystalline structures in the cytoplasm, with blunt, or splinter like ends. In maturing cysts, these crystalline masses fragment into separate particles and the chromatoid bodies tend to disappear (Chavez-Munguia et al., 2003). Similarly, vesicles fusing with the plasma membrane of the cyst and appearing to deposit their fibrogranular material to the cyst wall, has been shown for *E. histolytica* cysts (Chavez-Munguia and Martinez-Palomo, 2011). This process is contributed to a growing exocyst and incomplete cyst formation.

SEM revealed pronounced polygonal edges at the exterior surface of the smaller cysts, with a size of ~4 µm (**Figures 2E, F**). Polygonal edges were not reported for *E. histolytica* cysts, which are spherical in shape (Aguilar-Diaz et al., 2010; Chavez-Munguia and Martinez-Palomo, 2011). Instead, a polygonal shape with edges converging at putative osteooli and operculae resembled the cysts of *A. lugdunensis* (Garajova et al., 2019). Such smaller cysts can represent a different state of maturity.

**Figure 3:**
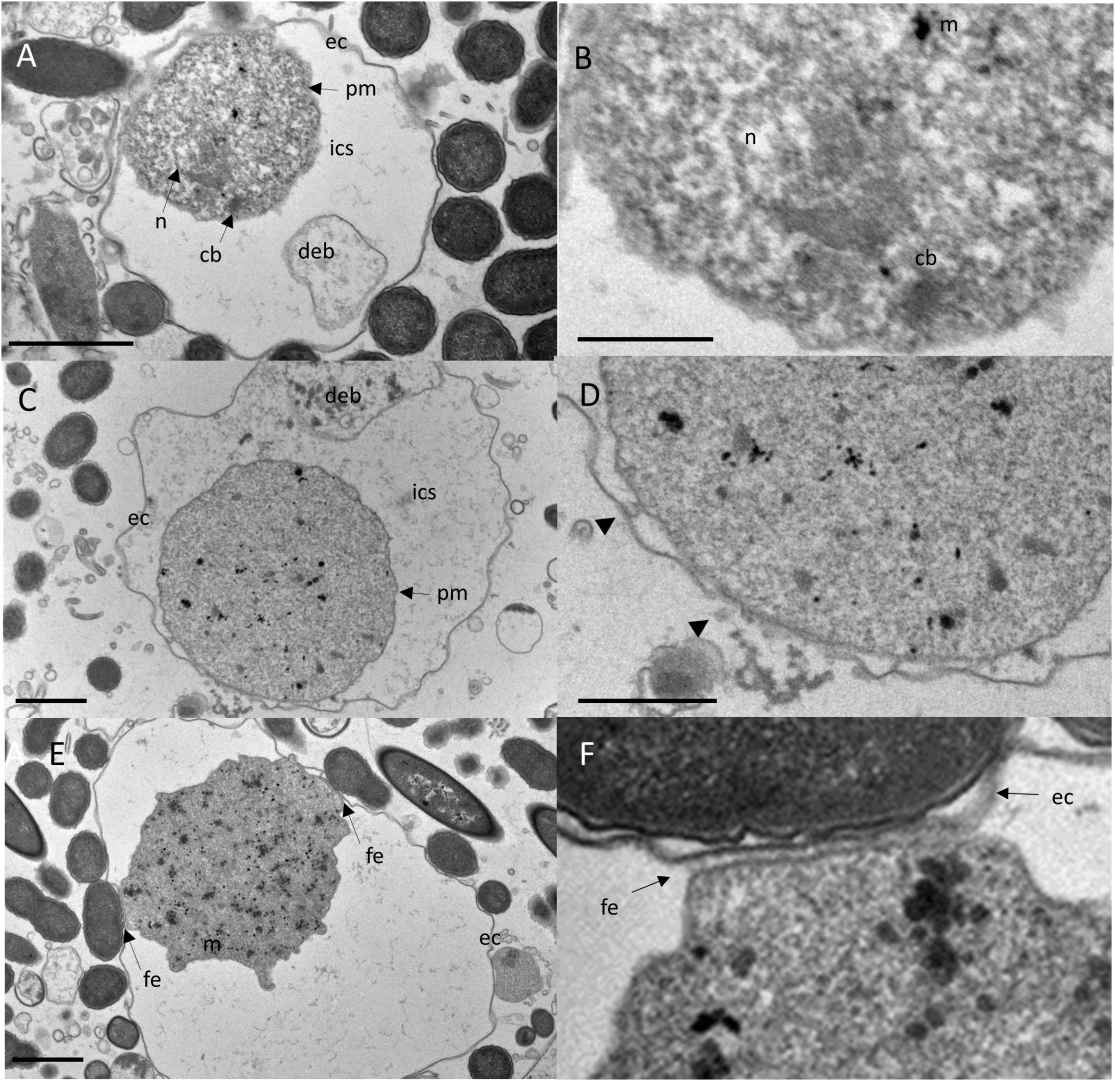
TEM reveals bidirectional growing of the cyst wall by vesicles released from the trophozoite and local fibrillary anchoring of the trophozoite to the exocyst. Upper panel: The trophozoite’s plasma membrane is separated from the exocyst by an electron-transparent intra cystic space (ics). Cell debris (deb) is encased within the ics **(A)**. Close-up view of A highlights an electron-dense capped mitosomes (m) and parallel electron-dense structures of a putative truncated chromatin body (cb) adjacent to the nucleus **(B)**. Scale bar = 5µm. Middle panel: A cyst developing the exocystic wall **(C)**. Close-up view of C, showing ec close at the pm with several vesicles free and fused with pm and ec (arrow heads), indicating a growing cyst wall and incompletion of encystment. The cyst wall is developing bidirectionally in close proximity to the trophozoite **(D)**. Scale bar = 1µm. Lower panel: An encysted trophozoid with two flanking points where pm is closely apposed to ec **(E)**. Close-up view of C, showing the connection of the trophozoid to the cyst wall, which consists of an array of perpendicular fibrillar elements (fe) **(F)**. Scale bar = 1µm.

The plasma membrane of the encysted trophozoids was wrinkled and in many cysts an electron-permeable intracystic space clearly separated the trophozoid from the cyst wall, with few flanking points where the plasma membrane and the inner side of the cyst wall were in close contact (**Figure 3A**). This observation was similar to that of *E. invadens* and *A. lugdunensis*, where in younger cysts (12 hour encystation for *E. invadens*), the cytoplasmic mass can be seen separate from the plasma membrane and in some cells, depending on the plane of section, the trophozoite appears to be completely separated from the cyst wall or to detach almost completely from the membrane (Garajova et al., 2019; Singh et al., 2021).

We also observed putative electron-dense, capped mitosomes and putative chromatin bodies adjacent to the nucleus, characterized by parallel electron-dense structures (**Figure 3B**). These structures resembled those of *A. lugdunensis* (Garajova et al., 2019) and other protists unrelated *Entamoeba* such as *Tritricamonas* (Tovar et al., 2003). Within the intra-cystic space at the area where the cytoplasmic membrane adhered to the exocyst, in some cells, we observed vesicles of 8-10 nm diameter, which fused with the plasma membrane and the exocyst (**Figures 3C and D**). Similarly, vesicles fusing with the plasma membrane of the cyst and appearing to deposit their fibrogranular material to the cyst wall, has been shown for *E. histolytica* cysts (Chavez-Munguia and Martinez-Palomo, 2011). This process is contributed to a growing exocyst and incomplete cyst formation. In some cysts, the areas where the trophzoites contacted the cyst wall showed numerous, perpendicular thin filaments that connected the plasma membrane with the cyst wall (**Figures 3E, F**). We hypothesize that these are areas with putative ostioles, small openings through which the trophozoid could escape from the cyst. As shown by TEM, these complexes were covered by flat structures, that may serve as operculae to open the cyst during excystation of the trophozoite. Ostioles and operculae have, to our knowledge, not been described for *Entamoeba* species before. However, they are commonly observed in other amoeba, like *Acanthamoeba* (Garajova et al., 2019).

**Figure 4:**
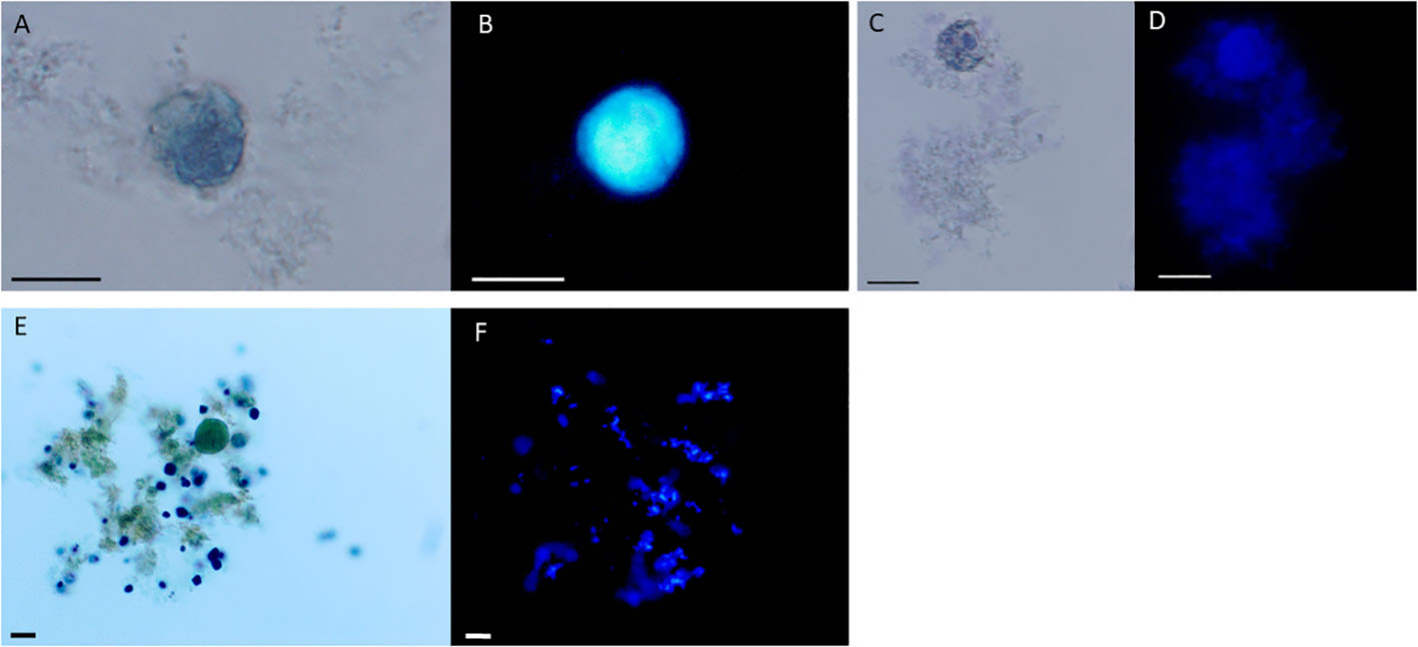
*Entamoeba gingivalis* cysts but not trophotoites show chitin walls by calcofluor white staining. *E. gingivalis* cysts were incubated with amoxycillin and metronidazole for 8 days and subsequently stained with Lugol`s iodine and calcofluor white **(A-E)**. *E. gingivalis* cysts under light microscopy without UV **(A, C)**. The cyst walls show blue fluorescence under UV, indicating the presence of chitin **(B, D)**. As a control, 2 days old *E. gingivalis* cultures, which were not treated with amoxycillin and metronidazole, were stained with Lugol`s iodine **(E)** and calcofluor white **(F)**. The trophozoite does not show blue fluorescence under UV, indicating absence of chitin. Scale bar = 10 µm.

A major scaffolding component of the cyst wall of *Entamoeba* species like *Entamoeba histolytica*, *Entamoeba dispar* and the reptilian parasite *Entamoeba invadens* is chitin, a homopolymer of beta- (Eke et al., 2015; Bao et al., 2020)-linked N-acetyl-D-glucosamine (Campos-Gongora et al., 2004). To visualize chitin at the whole cyst level and to give direct evidence that the cyst wall like structures observed by TEM and SEM have biochemical characteristics of *Entamoeba* cyst walls, we performed chitin staining using the common polysaccharide marker calcofluor white. We found that calcofluor white fluorescence reflected chitin content in the walls of amoeba that were treated with Amoxycillin and Metradinazole for 8 hours, but amoeba cultured for 2 days did not show calcofluor white fluorescence (**Figures 4A-F**). Taken together, our observations demonstrated that *E. gingivalis* is capable to develop cysts after exposure to antibiotics.

### *E. gingivalis* trophozoites do not develop cyst-like structures without antibiotics supplementation of *in vitro* cultures

**Figure 5:**
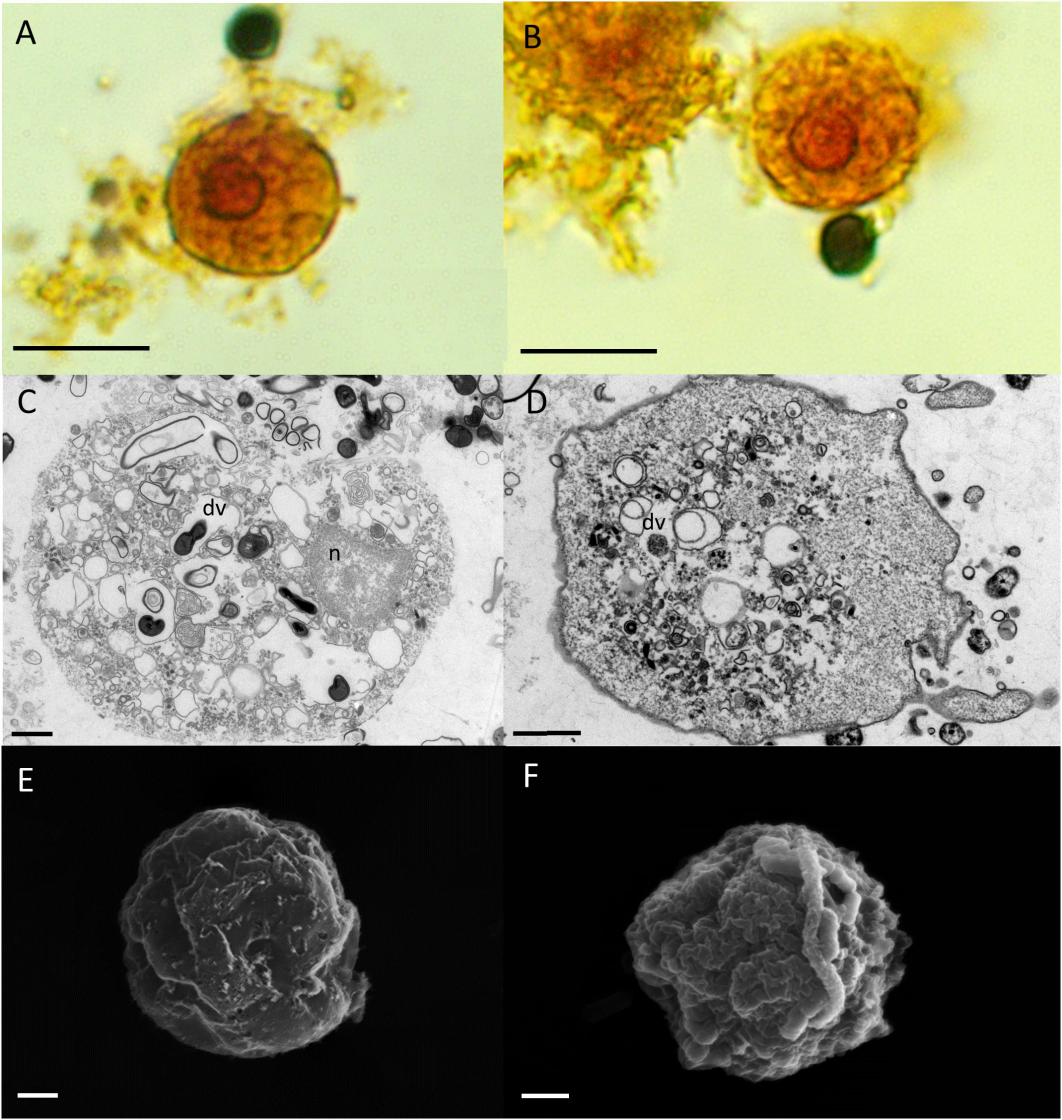
*E. gingivalis* cultured for 10 days in antibiotic free axenic medium are characterized by granulose wrinkled walls, the lack of pseudopodia and numerous digestive vacuoles. Upper panel: Under the light microscope, lugol’s iodine stained trophozoites are round shaped and show no pseudopods. The granulous cytoplasma and the nucleus with peripheral chromatin and a central karyosome is visible **(A, B)**. Scale bar = 10 µm. Middle panel: TEM reveals numerous digestive vacuoles (dv) in the cytoplasma of the round shaped trophozoites **(C, D)**. Scale bar = 1 µm. Lower panel: SEM reveals a wrinkled unstructured surface of the round shaped trophozoites with neither pseuodops nor digipods and filipods **(E, F)**. Scale bar = 1 µm.

We also observed that 10 days old *Entamoeba* cultures that were not treated with Amoxycillin and Metradinazole, also differed in shape from the active trophozoites, which we generally found in 2 days old cultures. A rounded spherical shape characterized many untreated trophozoites from *in vitro* cultures after 10 days (**Figure 5**). Light microscopy showed that the plasma membranes had irregular contact to a putative cell wall, which was occasionally separated by a transparent space. In these cells from 10 days old cultures, we also did not observe pseudopods and filamentous structures such as digipodes and filipodes that are characteristic for trophozoites. SEM additionally revealed smoother outer surfaces compared to the outer membrane surfaces of trophozoites from 2 days old cultures. However, we did not find polygonal edges and osteole like structures observed in cultures treated with Amoxycillin and Metradinazole. Moreover, unlike that seen in antibiotic treated 10 days old cultures, TEM revealed that the cytoplasma of untreated amoeba of the same culture age was heterogeneous and it contained, similar to trophozoites from young cultures, numerous vacuoles. These characteristics implied that the amoeba in 10 days old untreated cultures were, if any, cysts at different stage of maturity. Likewise, *E. invadens* cysts show many vesicles >12 hours after encystation and mature *E. histolytica* cysts also contains abundant vesicles (Chavez-Munguia et al., 2003).

## Discussion

By using antibiotics in combination and concentrations identical to those commonly prescribed antibiotic drugs for periodontal diseases (“van Winkelhoff cocktail”), we induced cyst formation of *E. gingivalis* trophozoites. Over the course of antibiotic exposure, the trophozoites became smaller, changing shape into a dense vacuole-free cytoplasm that was eventually completely enveloped by a chitin containing exocyst. At this stage of encystation, the plasma membranes of the trophozoites were clearly separated by inter-cystic spaces.

This is the first time that cyst stages have been observed in *E. gingivalis.* The reason for this could be that *E. gingivalis* trophozoites do not form cysts in the inflamed periodontal pocket, which is their preferred ecological niche (Bao et al., 2020). Another reason why *E. gingivalis* encystation has not been observed so far could be that unfavorable culture conditions such as sudden and prolonged exposure to aerobic conditions after removal from anaerobic periodontal pockets may not leave enough time for encystation, leading to death of strictly anaerobic *E. gingivalis* trophozoites within *in vitro* cultures. Perhaps this also explains the general difficulties in maintaining life *E. gingivalis in vitro* cultures collected from subgingival plaque.

Although we observed numerous characteristics of amoebic cysts, we did not observe multinucleated cysts, which are formed during the natural life cycle of *E. histolytica* (Orozco et al., 1988). In the natural environment, nuclear division of *E. histolytica* during the encystation process takes place in the absence of cytokinesis and the single nucleated trophozoite transforms into a multi-nucleated dormant cyst (Das and Lohia, 2002). Here, the function of the mature cysts that are released into the external environment from the host is to enable dissemination of the anaerobic amoeba within an aerobic environment. Therefore, development of a multi-nucleated dormant cyst is part of cell proliferation during the life cycle of *E. histolytica* and the pre-formed multiple nuclei allow rapid completion of cytokinesis after the amoeba re-gained a favorable environment in a new host. However, in *E. gingivalis*, we did not observe multi-nucleated cysts. This implies multiple nucleated cysts are not part of the proliferative life cycle of *E. gingivalis* (Singh et al., 2021). Instead, in our experiments, exposure of *E. gingivalis* to noxious antibiotics induced cyst formation. It is conceivable that the trophozoites built cysts for protection instead of proliferation. Notably, cysts of *E. histolytica* also show different nuclei numbers *in vitro* (Aguilar-Diaz et al., 2010) and also *in vivo* (Singh et al., 2021). Future studies are required to determine if *E. gingivalis* forms a cyst for reproduction in the natural life cycle.

Another limitation of our study is that we induced cyst formation *in vitro* but not *in vivo*. Future studies will test if therapeutic application of the “van Winkelhoff-Cocktail” or other antibiotics also induce cyst formation in the natural habitat of *E. gingivalis*. In this clinical context, cysts would entail serious difficulties for the elimination of *E. gingivalis*, especially if encystation takes place in deep periodontal pockets or even within gingival tissue. Here, encystation may also relate to the current epidemic of periimplantitis, a destructive inflammatory process that is triggered by the presence of dental implants (Heyman et al., 2020), during which gingival tissues surrounding the implant become inflamed, leading to alveolar bone loss over time. Moreover, cyst formation may also explain why sometimes, established treatment approaches fail to stop inflammatory periodontal tissue destruction in periodontitis and periimplantitis. Conversion into dormant cysts to outlast therapeutically measures such as antibiotic treatment may allow the amoeba to survive and to recolonize the habitat. This would require subsequent excystation of the trophozyte, a process that has already been described for *Entamoeba invadens* cysts (Mitra et al., 2010), but which was not addressed in the current study.

In summary, the current study provides evidence that the oral eukaryotic parasite *E. gingivalis* is capable of encystation after periodontal antibiotic treatment. This ability may allow this pathogen to survive periodontal therapy and recolonize periodontal or implant pockets. Encystation can also have implications for dissemination and inter-individual infections. In the future, elucidation the processes involved in cyst formation could help to develop new treatment approaches for periodontitis, e.g. by silencing the encystation pathway.

## Data availability statement

The original contributions presented in the study are included in the article/supplementary material. Further inquiries can be directed to the corresponding author.

## Ethics statement

The studies involving human participants were reviewed and approved by the local ethics committee of the Charité - Universitätsmedizin Berlin. The patients/participants provided their written informed consent to participate in this study.

## Author contributions

CB and AS contributed to data collection, interpretation, conception and design of the study and wrote the manuscript. AA and HD contributed to data collection. TS contributed to data collection and interpretation. All authors contributed to the article and approved the submitted version.
